# Effects of energy drinks on fertility, blood parameters and perinatal physiology in female rats

**DOI:** 10.1590/1980-220X-REEUSP-2026-0353en

**Published:** 2026-08-31

**Authors:** Halime Aydemir, Fatma Ergün, Ayşegül Turan

**Affiliations:** 1 Kirsehir Ahi Evran University, Faculty of Health Sciences, Department of Midwifery. Kirsehir, Türkiye.; 2 Kirsehir Ahi Evran University, Faculty of Health Sciences, Department of Nutrition and Dietetics, Kirsehir, Türkiye.; 3 Kirsehir Ahi Evran University, Faculty of Health Science, Department of Nursing. Kirsehir, Türkiye.

**Keywords:** Caffeine, Energy Drinks, Fertility, Perinatal Care, Rats, Cafeína, Bebidas Energéticas, Fertilidade, Assistência Perinatal, Ratos

## Abstract

**Objective:**

The increasing consumption of energy drinks, especially among children and young people, in recent years raises concerns. This study was aimed at investigating the effects of energy drink consumption on fertility, blood parameters, and perinatal physiology in female rats.

**Method:**

Three groups were formed. Of the groups, one was the control group and the other two were the experimental groups, namely 10% ED group and 20% ED group. In each group, there were 9 rats (3 rats per cage, 3 breeding cages per group). The rats in the control group were given only tap water, the rats in the 10% ED group were given a mixture including 10% energy drink and 90% tap water, and the rats in the 20% ED group were given a mixture including 20% energy drink and 80% tap water.

**Results:**

Pre-pregnancy energy drink consumption increased water and feed intake in female rats; however, it still increased fluid intake but reduced feed intake during pregnancy. The difference between groups was statistically significant (p < 0.05). Energy drink consumption affected hematological parameters depending on the dose, increased serum creatinine levels, affected total cholesterol levels according to consumption, increased total protein levels, and decreased alkaline phosphatase levels. Consumption of energy drinks also reduced the number of offspring and postnatal weight gain in a dose-dependent manner.

**Conclusion:**

It was concluded that energy drinks had effects on female rats during the prenatal and perinatal periods. To investigate the potentially concerning effects of energy drink consumption and to identify possible long-term health problems it might cause, further animal and human studies should be conducted. These results provide critical biological evidence to help midwives, women’s health nurses, and public health nurses design evidence-based premarital counseling and prenatal education programs in which the risks of energy drink consumption on fertility, maternal nutrition, and neonatal development are addressed.

## RESUMO

**Objetivo:**

O aumento do consumo de bebidas energéticas, especialmente entre crianças e jovens, nos últimos anos, suscita preocupações. Este estudo teve como objetivo investigar os efeitos do consumo de bebidas energéticas sobre a fertilidade, os parâmetros sanguíneos e a fisiologia perinatal em ratas.

**Método:**

Foram formados três grupos. Desses, um era o grupo controle e os outros dois eram os grupos experimentais, a saber, o grupo 10% BE e o grupo 20% BE. Em cada grupo, havia 9 ratas (3 ratas por gaiola, 3 gaiolas de reprodução por grupo). As ratas do grupo controle receberam apenas água da torneira; as ratas do grupo 10% BE receberam uma mistura composta por 10% de bebida energética e 90% de água da torneira; e as ratas do grupo 20% BE receberam uma mistura composta por 20% de bebida energética e 80% de água da torneira.

**Resultados:**

O consumo de bebida energética antes da gestação aumentou a ingestão de água e ração nas ratas; no entanto, durante a gestação, ainda aumentou a ingestão de líquidos, mas reduziu a ingestão de ração. A diferença entre os grupos foi estatisticamente significativa (p < 0,05). O consumo de bebidas energéticas afetou os parâmetros hematológicos dependendo da dose, aumentou os níveis séricos de creatinina, afetou os níveis de colesterol total de acordo com o consumo, aumentou os níveis de proteína total e diminuiu os níveis de fosfatase alcalina. O consumo de bebidas energéticas também reduziu o número de filhotes e o ganho de peso pós-natal de maneira dependente da dose.

**Conclusão:**

Concluiu-se que as bebidas energéticas tiveram efeitos em ratas durante os períodos pré-natal e perinatal. Para investigar os efeitos potencialmente preocupantes do consumo de bebidas energéticas e identificar possíveis problemas de saúde a longo prazo que elas possam causar, devem ser realizados mais estudos em animais e em seres humanos. Esses resultados fornecem evidências biológicas essenciais para ajudar parteiras, enfermeiras especializadas em saúde da mulher e enfermeiras de saúde pública a elaborar programas de aconselhamento pré-matrimonial e educação pré-natal baseados em evidências, nos quais sejam abordados os riscos do consumo de bebidas energéticas para a fertilidade, a nutrição materna e o desenvolvimento neonatal.

## INTRODUCTION

Humans continuously develop and change throughout their lives. During this process, they influence their environment, and are influenced by it in return. For humans, in particular, the early stages of life such as childhood, adolescence, and youth are of great importance. Inadequate and unbalanced nutrition during these periods can affect later stages of life. Healthy eating during the early phases of life may help reduce potential negative outcomes. In recent years, changes in dietary patterns have led to a diversification in the foods and beverages we consume. Nutrition in humans ranks among the key factors that directly affect their reproductive health and fertility by influencing the endocrine system^(1)^.

Beverages make up a significant part of our daily diet. The most widely consumed liquid on earth is water. The body fluid requirement of an individual can be calculated as 1 mL of fluid per calorie consumed per day or 30 mL of fluid per kilogram. The European Food Safety Authority (EFSA) reports that the reference value of total fluid intake is 2.5 L for men and 2 L for women^(2)^. The variety and number of liquids used as beverages are increasing day by day.

As a result of the developments in the food sector, there is a significant increase in caffeine consumption and an increase in the demand for caffeinated beverages^(3)^. The increase in both the quantity and variety of daily fluid intake has been associated with the emergence of various health problems. Among these health issues, infertility-related disorders hold a significant position. In several studies, it has been indicated that reducing or avoiding caffeine consumption may enhance fertility in both women and men^(4)^. Caffeine is present in high amounts particularly in coffee, tea, and energy drinks, whose consumption has increased in recent years^(5)^. Globally, the consumption of energy drink (ED) has been steadily rising. These beverages are commonly consumed by adolescents and young adults. It is reported that more than 50% of EDs are consumed by those in these age groups^(6)^. Additionally, in a study conducted by Seifert at el., (2011), it was reported that 31% of adolescents aged between 12 and 17 years, and 34% of young adults aged between 18 and 24 years regularly consumed EDs^(7)^.

Energy drinks differ from other caffeine-containing beverages due to their chemical composition. The primary ingredients of these beverages include caffeine, taurine, guarana, sugar, sodium, and vitamin B6^(8)^. In addition, certain energy drinks produced by specific manufacturers also contain components such as glucuronolactone, ginseng, and ginkgo biloba^(9)^.

In several recent studies, the short- and long-term effects of energy drinks on the cardiovascular and central nervous system have been investigated. As reported in these studies, energy drinks cause various metabolic disorders such as high blood pressure, cardiovascular diseases, kidney disorders and sleep disorders in humans, especially in young people^(10)^. Energy drinks are known to increase fatigue after a certain period of consumption and increase consumption of energy drink when combined with other beverages. It has also been reported to reduce water consumption and salivation and trigger dental erosion. It is also known to cause nervous disorders, coronary artery spasm and cardiac arrest in young people due to excessive consumption^(11)^. In addition, in experimental studies conducted with animal models, it has been reported that ED consumption causes histopathological disorders in various organs including the brain, liver, kidneys and heart, leads to obesity, and increases cholesterol and blood glucose levels^(12)^.

In recent years, the increasing prevalence of infertile disorders among young people has led to an understanding of the importance of the issue and an investigation of the underlying causes. Given that midwives and nurses are among the primary health professionals who provide health education and counseling for women of reproductive age, understanding the physiological effects of energy drinks is essential for developing effective patient education strategies. In a review of the literature, it was revealed that the number studies in which the effects of energy drinks on pregnancy and fertility are investigated is very few. The effects of ED consumption during pregnancy are not yet clear. In the present study, an experimental animal model was established using newly weaned rats. With this model, it was aimed to investigate the effects of energy drinks on fertility, blood parameters and perinatal physiology in female rats.

## METHOD

The 20-day-old female rats (Wistar albino) used in the study were bred in Kirsehir Ahi Evran University Faculty of Medicine Animal Experiments Unit. The rats were kept in an artificial 12-hour light, 12-hour dark cycle (lights on between 7:00 a.m. and 7:00 p.m.) at a constant temperature of 22 ± 2 °C and 65% relative humidity. The rats used in the study were fed ad libitum. Commercially produced (OPTIMA) Standard laboratory rodent diet (24% crude protein, 3.94% crude cellulose, 5.08% crude fat, 8.8% crude ash, 1.44% lysine, 0.61% methionine, 1.14% calcium, 0.89% phosphorus, 0.28% sodium) was used in feeding the rats. Before the experiments were started, the animal use protocol was approved by the local ethics committee for animal experiments at Kirsehir Ahi Evran University (Date – 06/03/2025, No: 5/7). All the methods and details used in the study were carried out in accordance with the criteria specified in the ARRIVE guidelines. Furthermore, in all the procedures applied to live animals in the study, it was complied with the Regulation on the Welfare and Protection of Animals Used for Experimental and Other Scientific Purposes of the Ministry of Food, Agriculture and Livestock of the Republic of Turkey^(13)^.

Procedures established by the Kirsehir Ahi Evran University Local Ethics Committee were used to monitor the health and welfare of the animals used in the study. For this purpose, the animals used in the study were monitored daily until the end of the study for changes such as weight loss, death, behavioral changes, decreased food/feed and water intake, infection, abscess, dehydration, malnutrition, general weakness, diarrhea, constipation, ileus, convulsions, and coma^(13,14)^.

No signs of distress or illness were observed in any animal during any period of the study, and no early euthanasia criteria were applied. In cases such as traumatic disorders caused by human reasons, weight loss of more than 25% of body weight, inability to walk properly, inadequate food/feed and water intake, or significantly decreased response to stimuli as determined by the Kirsehir Ahi Evran University Local Ethics Committee’s directive (14) regarding the procedure of “Criteria for exclusion of animals from the research protocol”, if the Veterinarian deems it appropriate, the animal or animals are removed from the study and euthanized by administering high doses of xylazine (10 mg/kg live weight intraperitoneally) and ketamine (60 mg ketamine hydrochloride/kg live weight, intraperitoneally).

### Energy Drink Ingredients

Red Bull /Sugarfree^®^ brand sugar-free ED, which is widely consumed by young people, was used in the study. This ED is sold in 250 mL cans and contains caffeine (150 mg/L), taurine (800 mg/L), vitamins (Nisain (8 mg/100 mL), Pantetonic Acid (2 mg/100 mL), B6 (2 mg/100 mL), B12 (2 µg/100 mL), flavor and thickener (Xanthan gum), colorant (Plain Caramel, Riboflavin) and phenylalanine. The reason why sugar-free ED was preferred was the effects related to carbohydrate consumption and the thought that these effects may mask other results. The amount of ED to be given to the rats was calculated by considering that an adult weighing 65 kg could meet 10% and 20% of the total amount of liquid that should be consumed if he/she drinks one can of ED (250 mL) and two cans (250 mL + 250 mL), respectively per day^(15)^. The ingredients of energy drinks vary little between companies. Therefore, energy drinks generally contain similar ingredients (caffeine, taurine, glucuronolactone, sugar, B vitamins, and plant extracts (ginseng, guarana, yerba mate, and green tea extracts).

### Study Groups

Based on similar published studies, a power analysis was conducted using G*Power and the MINITAB statistical software package^(16)^. As a result of this analysis, it was decided to include a minimum of nine animals per group to achieve adequate statistical power, resulting in a total of 27 animals^(16,17)^. Three groups were formed with 9 rats in each group (3 rats in 1 cage, three breeding cages in each group) (a total of 27 rats). While only tap water was given to the rats in the control group (C), a mixture including 90% tap water and 10% ED was given to the rats in the 10% ED group, and a mixture including 80% tap water containing and 20% ED was given to the rats in the 20% ED group ad libitum throughout the study (from the first day of the study until the rats gave birth).

### Experimental Procedure

**Figure 1:**
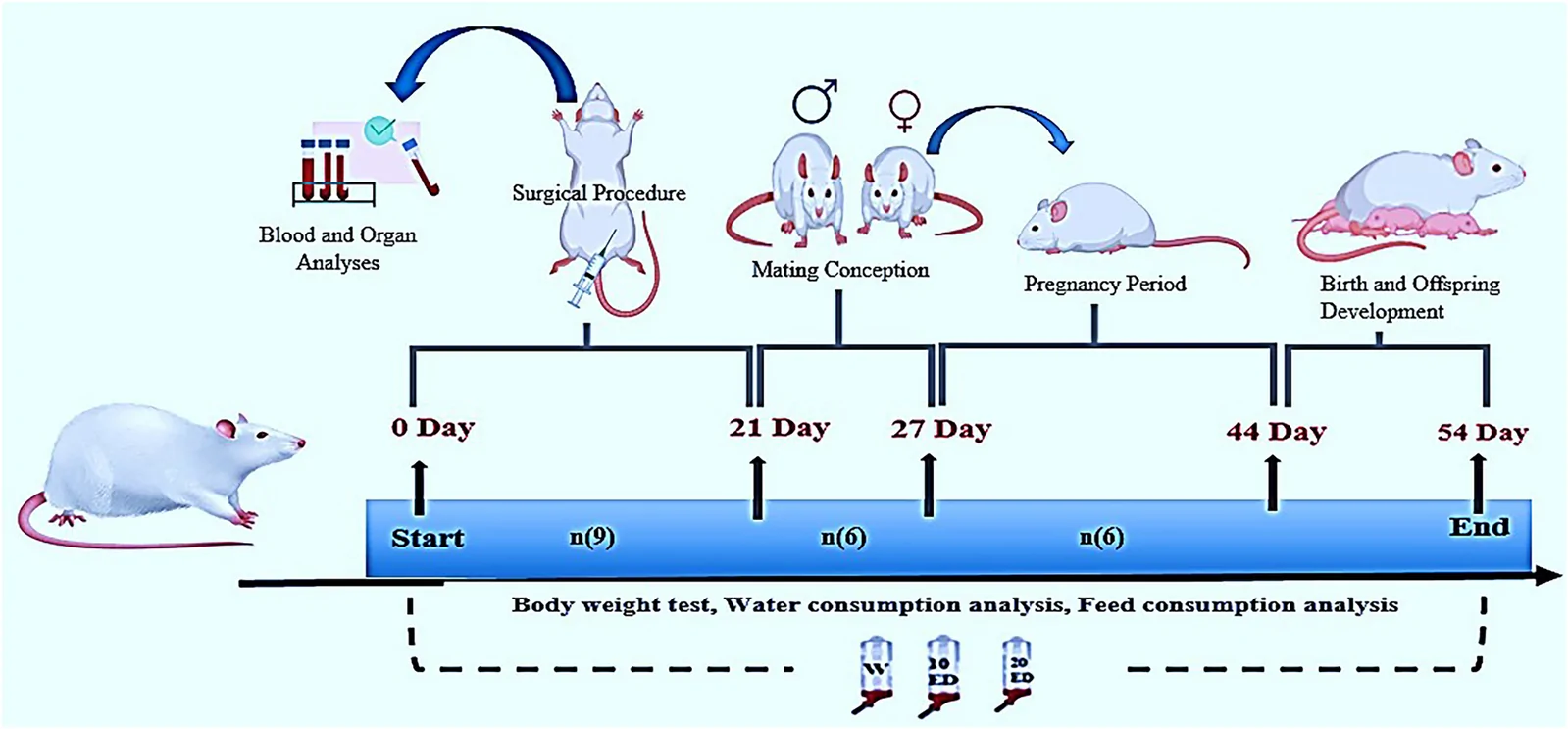
General chart of the study design.

The experiment was carried out in two phases over a total of 54 days. The first phase was 21 days and ED was given to the groups at the determined rates. At the end of 21 days, the abdominal regions of 3 rats with a body weight of 113.33 ± 1.52 g from each group were incised under general anesthesia with xylazine (10 mg/kg bw intraperitoneal) and ketamine (60 mg ketamine hydrochloride/kg bw, intraperitoneal) and under asepsis and antisepsis conditions. The rats were bled through the abdominal aorta before death. In addition, utero-ovarian, kidney, liver, heart and brain tissues were removed for the calculation of organ indices. In the second phase of the study, the remaining 6 rats in each group were allowed to become pregnant. Two female rats and one male rat were kept in mating cages for 6 days. Then, female rats were placed in breeding cages (425*265*150/800 cm^2^) with 2 rats in each cage. All rats were kept in these cages until the end of the study (Figure 1).

### Analyses

#### Physical Analyses

***Feed and water consumption and body mass index:*** The amounts of feed and water consumed by the rats were measured at 3-day intervals and the amounts of water (mL/100g BW) and feed (g/100g BW) consumed by the rats were determined at the end of the study. Body weight (BW) (NECK WT-NF Precision Scale) and body length (naso-anal length) (cm) (the distance from the nasal bone to the anus when the rats were lying on their stomach) were measured. Lee mass index of the study groups were calculated on days 0, 10, 21, 40 using the following formula^(18)^.


$$\text{Lee mass index}=\frac{\sqrt[{3}]{\text{Body weight (g)}}}{\text{Body length (cm)}}\times 1000$$

### Motor Function Analysis

***Kondziela’s inverted screen test (KIST):*** Kondziela’s inverted screen test is a muscle strength test using all four limbs. For this purpose, a 30 × 30 cm plastic apparatus with 2 × 2 cm grid holes specially designed for the study was used. First, the rats were placed in the center of the apparatus. Then they were inverted from a height of 40–50 cm. When the head of the rat was tilted downwards, a stopwatch was started and the hanging time of the rats was measured.

***Weight test (WT):*** Forelimb muscle strength of rats was measured with this test. Lead weights (gr) attached to a ball made of knitting string were used as weight apparatus^(19)^. Each rat was held by the middle of its tail and lowered down to grasp the ball. After the rats grasped the ball with their front paws, they were lifted upwards to lift the weight hanging on the ball together with the ball. If the rat dropped the weight in less than 3 seconds, a second trial was performed after 10 seconds. If it failed three times, this trial was terminated and the maximum weight the rat could lift was determined.

### Blood and Serum Analyses

On the 21st day of the study, blood samples collected via the abdominal aorta were taken into Ethylenediaminetetraacetic acid (EDTA) tubes for hematological analysis. To obtain serum from the blood samples collected for biochemical parameters, a total of 1.5 ml of blood was centrifuged (2500 rpm, 10 min, 4°C). Serum concentrations of creatinine (CRE), calcium (Ca), gamma-glutamyl transferase (GGT), total cholesterol (TCHO), total protein (TP), alkaline phosphatase (ALP), albumin/globulin ratio (ALB/GLOB), and glucose (GLU) in female rat serum samples were determined using commercial assay kits on a FUJIFILM DRI-CHEM NX600 (Hasvet®) analyzer. Additionally, using the Mindray BC-30 Vet (hasvet®) device, analyses of hematological parameters from EDTA blood samples were performed, including white blood cell (WBC), lymphocyte (LYM), monocyte (MID), granulocyte (GRAN), neutrophil lymphocyte ratio (NLR), platelet-lymphocyte ratio (PLR), red blood cell (RBC), hemoglobin (HGB), hematocrit (HCT), mean corpuscular volume (MCV), mean corpuscular hemoglobin (MCH), mean corpuscular hemoglobin concentration (MCHC), red blood cell distribution width (RDW-CV), platelet count (PLT), mean platelet volume (MPV), platelet distribution width (PDW), procalcitonin (PCT), platelet large cell ratio (PLCR), and platelet large cell coefficient (P-LCC).

### Anatomical Analyses

On day 21 of the study, the utero-ovarian complex, heart, liver, brain, and kidneys obtained from rats subjected to surgical procedures were rapidly dissected. Connective tissue and fascia were removed, and any surface fluids on the organs were dried off using filter paper. Subsequently, the organs were weighed. Organ index were then calculated using the following formula^(20)^.


$$\text{Organ Index (\%)}=\frac{\text{Organ weight (g)}}{\text{Total body weight (g)}}\times 100$$

### Analysis of Fertility and Offspring Data

In the second phase of the study, the female rats that had been separated from the male ones after mating and housed in breeding cages were monitored for parturition. At the end of the observation period, the number of females that gave birth within each group, the number of offspring, birth weight of the pups, and daily weight gain of the offspring until day 10 were recorded.

### Adverse Events

No complications related to energy drink consumption were identified in the rats in this study. No clinical or pathological signs were observed in the animals during the study period.

### Statistical Analyses

The data obtained from the study were analyzed using the SPSS (Statistical Package for the Social Sciences), version 22. Initially, the effects of ED consumption on the physical and physiological characteristics of young, pregnant, and offspring rats were determined using one-way analysis of variance (ANOVA). When there were significant differences between the groups, Duncan’s multiple comparison test was applied to pinpoint the specific treatment or treatments that led to these variations. For all statistical evaluations performed in this study, a p-value of less than 0.05 (p < 0.05) was regarded as statistically significant.

### Ethics Approval and Consent to Participate

Permission was obtained from the local ethics committee of Kirsehir Ahi Evran University Animal experiments dated March 06, 2025 and numbered 5/7. All methods and details used in the study were carried out in accordance with the criteria specified in the ARRIVE guidelines. Procedures established by the Kirsehir Ahi Evran University Local Ethics Committee were used to monitor the health and welfare of the animals used in the study.

## RESULTS

In the study, daily feed and water consumption of pre-mating female rats and pregnant rats in each group was calculated. Differences between the groups were statistically significant at the p < 0.05 level. The highest water intake was observed in the 10% ED groups. The highest feed consumption was recorded in the 20% ED group among pre-mating female rats, and in the C group among pregnant rats. In the study, the body weights and body lengths of the female rats in the groups were measured on days 0., 10., 21., and 40., and their lee mass index values were calculated. Differences in the lee mass index values between the groups were significant at the level of p < 0.05 (Table 1). It was determined that the consumption of ED before and during pregnancy increased the index values.

**Table 1:** Daily feed and fluid consumption and lee mass index findings of the groups – Kirsehir, Türkiye, 2026.

Groups	Daily feed and fluid consumption amounts of the groups						
	Feed consumption of pre-mating female rats (g/100 g BW/day)		Water/fluid consumption of pre-mating female rats (mL/100 g BW/day)		Feed consumption of pregnant rats (g/100 g BW/day)		Water/fluid consumption of pregnant rats (mL/100 g BW/day)
C	0.13 ± 0.51^b^		0.45 ± 0.04^b^		0.17 ± 0.05^a^		0.50 ± 0.03^b^
10% ED	0.14 ± 0.34^a^		0.58 ± 0.03^a^		0.16 ± 0.01^a^		0.54 ± 0.01^a^
20% ED	0.14 ± 0.80^a^		0.48 ± 0.03^b^		0.16 ± 0.01^a^		0.49 ± 0.01^b^
**Groups**	**Lee mass index values of the groups at different time intervals**						
	**0. day**		**10. day**		**21. day**		**40. day**
C	293.70 ± 6.98^a^		285.82 ± 0.45^b^		289.04 ± 6.16^b^		288.00 ± 6.03^b^
10% ED	292.69 ± 2.43^a^		296.55 ± 0.61^a^		298.00 ± 2.84^a^		296.28 ± 5.73^a^
20% ED	290.40 ± 4.30^a^		295.98 ± 0.38^a^		297.13 ± 3.59^a^		294.80 ± 4.86^ab^

*: Differences between means indicated by the same letter are not significant at the p < 0.05 level; C: Control Group was given tap water; 10% ED Group was given a mixture including 10% energy drink and 90% tap water; 20% ED Group was given a mixture including 20% energy drink and 80% tap water.

**Figure 2:**
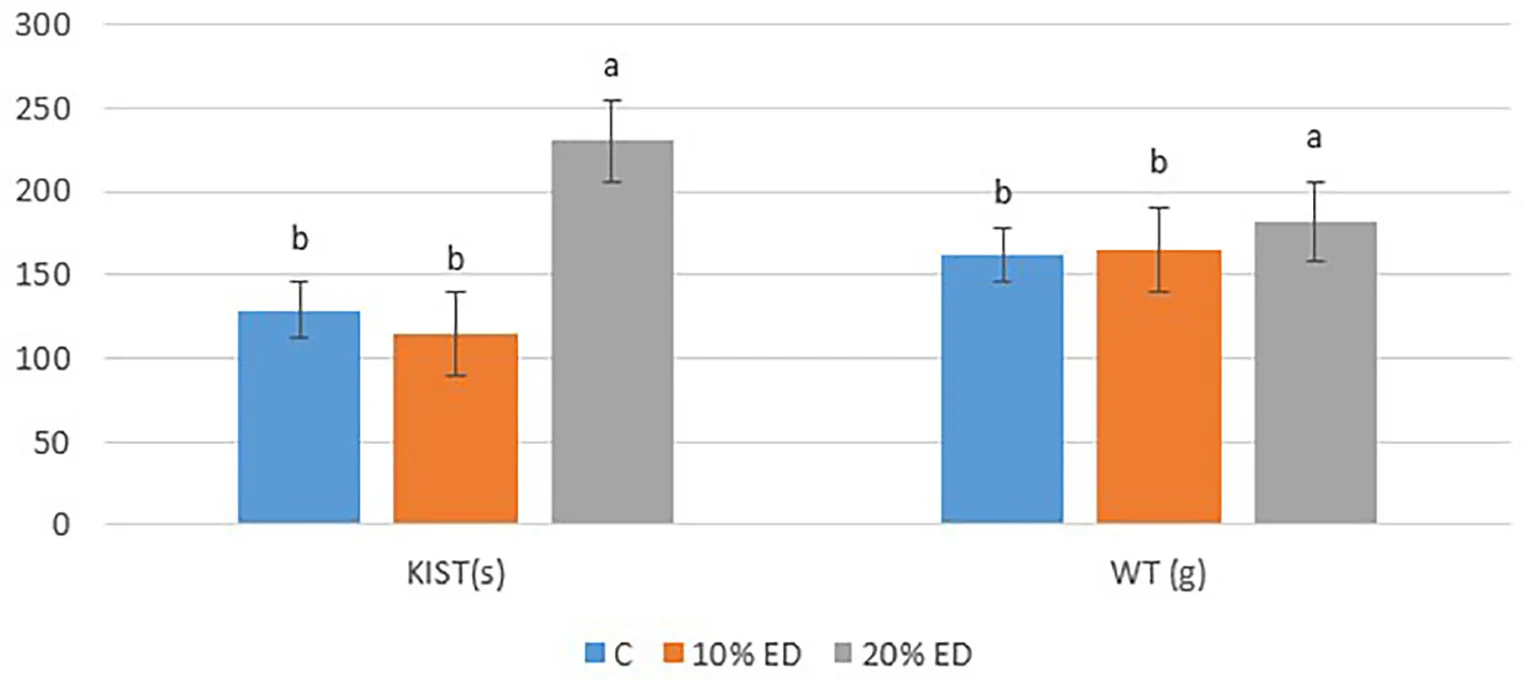
Results of the Kondziela’s inverted screen test and weight test applied to the groups.

KIST: Kondziela’s inverted screen test; WT: Weight Test; C: Control Group was given tap water; 10% ED Group was given a mixture including 10% energy drink and 90% tap water; 20% ED Group was given a mixture including 20% energy drink and 80% tap water.

In the study, the motor functions of the rats before pregnancy were evaluated using two paradigms. The muscle strength capabilities of the four limbs of the rats were measured with Kondziela’s inverted screen test. Differences between the groups were significant (p < 0.05). Additionally, the highest value in the weight test among the groups was observed in the 20% ED group (p < 0.05) (Figure 2).

On day 21 of the study, blood sera collected from the rats were analyzed. The differences between the groups in terms of CRE, TCHO, TP, ALP, ALB/GLOB, and GLU levels were statistically significant (Table 2) (p < 0.05). In the study, complete blood analyses were performed on pre-mating female rats, and hematological parameter results for each group were determined. Statistically significant differences were determined between the groups in terms of WBC, RBC, HGB, HCT, MCV, MCHC, RDW-CV, PLT, MPV, PCT, and P-LCC parameters (p < 0.05) (Table 2).

**Table 2:** Values of blood parameters of the groups – Kirsehir, Türkiye, 2026.

Parameters	Values of biochemical parameters of the groups			
	C	10% ED	20% ED	Reference value
CRE	0.28 ± 0.05^b^	0.31 ± 0.01^a^	0.33 ± 0.02^a^	–
Ca	4.36 ± 0.32^a^	4.70 ± 0.55^a^	4.83 ± 0.15^a^	–
GGT	10.66 ± 1.15^a^	11.33 ± 2.30^a^	10.33 ± 0.57^a^	–
TCHO	52.33 ± 4.04^ab^	56.66 ± 0.57^a^	50.33 ± 0.57^b^	–
TP	5.93 ± 0.28^b^	6.66 ± 0.11^a^	6.00 ± 0.20^b^	–
ALP	5.26 ± 0.57^a^	4.50 ± 0.10^ab^	4.13 ± 0.49^b^	–
ALB/GLOB	2.76 ± 0.57^a^	1.93 ± 0.20^b^	1.96 ± 0.11^b^	–
GLU	134.66 ± 2.30^b^	178.00 ± 8.66^a^	172.00 ± 15.58^a^	–
	**Values of hematological parameter of the groups**			
WBC (10^9^/L)	11.29 ± 1.38^ab^	10.59 ± 0.24^b^	13.16 ± 1.53^a^	1.90–13.80
Gran (10^9^/L)	3.16 ± 0.32^a^	2.40 ± 0.75^a^	3.74 ± 0.90^a^	0.39–4.60
Lym (10^9^/L)	**7.57 ± 1.08** ^a^	**7.24 ± 0.22** ^a^	**7.22 ± 1.76** ^a^	1.31–7.20
Mon (10^9^/L)	0.57 ± 0.02^b^	0.59 ± 0.06^b^	0.82 ± 0.15^a^	0.00–0.92
Gran (%)	0.26 ± 0.02^a^	0.28 ± 0.04^a^	0.27 ± 0.03^a^	0.120–0.460
Lym (%)	0.68 ± 0.03^a^	0.66 ± 0.03^a^	0.66 ± 0.03^a^	0.500–0.859
Mon (%)	0.050 ± 0.008^a^	0.051 ± 0.008^a^	0.055 ± 0.01^a^	0.000–0.103
RBC (10^12^/L)	6.10 ± 0.35^b^	**7.49 ± 0.50** ^a^	**7.09 ± 0.53** ^a^	4.82–9.80
HGB (g/Dl)	13.90 ± 0.17^b^	**16.73 ± 0.28** ^a^	**16.56 ± 0.75** ^a^	11.0–17.0
HCT (%)	39.00 ± 1.07^b^	**45.16 ± 0.40** ^a^	**45.36 ± 1.87** ^a^	27.0–46.0
MCV (fL)	64.10 ± 2.62^a^	58.73 ± 1.76^b^	62.50 ± 0.69^a^	50.0–67.0
MCH (pg)	22.86 ± 1.25^a^	21.70 ± 0.52^a^	22.26 ± 0.40^a^	16.0–25.0
MCHC (g/dL)	355.33 ± 6.42^b^	369.66 ± 2.51^a^	355.66 ± 2.30^b^	310–390
RDW-CV	0.15 ± 0.06^b^	0.11 ± 0.07^c^	0.17 ± 0.02 ^a^	0.110–0.150
RDW-SD (fL)	38.30 ± 1.87^b^	27.40 ± 1.21^c^	**42.56 ± 0.28** ^a^	25.0–40.0
PLT (10^9^/L)	872.33 ± 10.64^b^	1251.33 ± 2.88^a^	822.33 ± 40.47^b^	250–1400
MPV(fL)	6.63 ± 0.32^b^	6.43 ± 0.37^b^	7.23 ± 0.11^a^	5.1–8.2
PDW	15.30 ± 0.40^a^	14.96 ± 0.05^a^	15.30 ± 0.40^a^	12.0–17.5
PCT (mL/L)	5.96 ± 0.56^c^	**8.30 ± 0.09** ^a^	7.16 ± 0.56^b^	2.00–8.20
P-LCC (10^9^/L)	299.33 ± 27.13^a^	429.66 ± 11.54^a^	373.33 ± 13.27^a^	100–580
P-LCR	0.32 ± 0.02^c^	0.37 ± 0.02^b^	0.42 ± 0.09^a^	0.220–0.570

*Differences between means indicated with the same letter in each row are insignificant at p < 0.05 level; C: Control Group was given tap water; 10% ED Group was given a mixture including 10% energy drink and 90% tap water; 20% ED Group was given a mixture including 20% energy drink and 80% tap water; CRE: Creatinine; Ca: Calcium; GGT: Gama- transferase; TCHO: Total Cholesterol; TP: Total Protein; ALP: Alkaline Phosphatase; ALB/GLOB: Albumin/Globulin ratio; GLU: Glucose; WBC: White blood cell; LYM: lymphocyte; MON: monocyte; GRAN: granulocyte; RBC: Red blood cell; HGB: Hemoglobin; HCT: Hematocrit; MCV: Mean corpuscular volume; MCH: Mean corpuscular hemoglobin; MCHC: Mean corpuscular hemoglobin concentration; RDW-CV: Red blood cell distribution width; PLT: Platelet count; MPV: Mean platelet volume; PDW: Platelet distribution width; PCT: Procalcitonin; PLCR: Platelet large cell ratio; P-LCC: Platelet large cell coefficie.

On the 21st day of the study, organ weight indices of the rats subjected to surgery were determined. In the study, differences between brain, kidney and heart organ indices were insignificant (p < 0.05). However, differences between utero-ovarian and liver tissue indices were significant. At the end of the study, the number of offspring of pregnant rats that gave birth in the groups, their weights and the live weights of the offspring at the end of the 10th day were determined. The differences between the groups in terms of the number of offspring born and the average live weight values of the offspring at the end of the 10th day were significant (Table 3) (p < 0.05).

**Table 3:** Data on organ coefficients and offspring characteristics of the groups (%) – Kirsehir, Türkiye, 2026.

Groups	Data of the organ coefficient				
	Utero-ovarian index	Cardiac index	Liver index	Kidneys index	Brain index
C	0.44 ± 0.02^b^	0.41 ± 0.00^a^	4.69 ± 0.18^b^	0.95 ± 0.07^a^	0.95 ± 0.04^a^
10% ED	0.55 ± 0.02^a^	0.42 ± 0.00^a^	5.27 ± 0.23^a^	0.93 ± 0.06^a^	0.92 ± 0.13^a^
20% ED	0.37 ± 0.00^c^	0.40 ± 0.05^a^	4.38 ± 0.35^b^	0.10 ± 0.07^a^	0.98 ± 0.15^a^
**Groups**	**Data on the offspring of the groups**				
	**Number of offspring (pieces)**		**Birth weights (gr)**		**10th day offspring weights (gr)**
C	12.75 ± 0.95^a^		5.89 ± 0.42^a^		18.94 ± 0.41^a^
10% ED	11.75 ± 0.95^a^		6.24 ± 0.55^a^		18.62 ± 0.68^a^
20% ED	10.75 ± 1.50^b^		6.48 ± 0.74^a^		16.70 ± 0.83^b^

*Differences between means indicated by the same letter are not significant at the p < 0.05 level; C: Control Group was given tap water; 10% ED Group was given a mixture including 10% energy drink and 90% tap water; 20% ED Group was given a mixture including 20% energy drink and 80% tap water.

## DISCUSSION

In the study, an increase in body weight of rats was determined due to ED consumption. Adjene et al.^(21)^ reported that long-term ED consumption increased body weight in their study on rats. Mustafa et al.^(22)^ aimed to assess the side effects of EDs such as Red Bull and Red Strong on many physiological parameters and histological features of liver and kidney in male albino rats and found that body weight gain increased significantly in the long-term high ED groups. The results of the present study are consistent with the results of Adjene et al.’s^(21)^ study. On the other hand, in a rat study, it was reported that rats given ED for 140 days experienced significant body weight loss compared to the rats in the control group^(23)^. This result is different from that of the present study. It is thought that this difference was probably due to the difference in the duration of ED consumption of rats.

Although various standard criteria have been established for the detection of obesity in humans, obesity and body weight gain, including in animals, are detected using various methods such as Lee body mass index. In Lee body mass index, values greater than 310 are accepted as an indicator of obesity^(18)^. The values determined in the present study are below this value. The highest values were observed in the groups consuming EDs, suggesting that EDs may have the potential to cause obesity, and that the effect of caffeine and flavorings in EDs may also cause this situation. Caffeine can also cause an increase in food consumption due to insomnia^(24)^.

In the present study, muscular endurance and strength in the groups consuming ED were higher than were those in the control group, probably due the fact that the chemical components in EDs have an effect on this situation, because these chemicals in ED act as antioxidant protectors in regulating the working capacity of muscles, aerobic endurance and Ca^2+^ transport in muscles. They also play an important role as regulators of osmotic pressure in tissues^(25)^.

In the present study, it was determined that ED dose- dependent consumption increased the amount of serum CRE, which is a breakdown product of muscle metabolism, and activities such as excessive exercise and activity and high protein consumption increase this amount. In a 12-week study of rats consuming energy drinks, elevated liver enzymes, impaired renal function, and impaired lipid results were reported^(26)^. In another study, histopathological changes in kidney and liver tissues were reported in rats consuming EDs^(22)^. The results obtained in that study are consistent with those obtained in the present study.

In the present study, no correlation was detected between the groups in terms of the amount of ED consumption and serum TCHO level. The highest value was determined in the 10% ED group and the lowest value was determined in the 20% ED group. The differences between the groups can be explained by the fact that 10% ED consumption increases TCHO level by increasing acetyl-CoA which is necessary for cholesterol synthesis in the liver of female Wistar rats^(27)^. In the present study, ED consumption increased serum TP in the female rats. In a study, it was reported that ED consumption decreased TP levels in female rats, which may be due to impaired hepatic protein synthesis resulting from liver dysfunction^(28)^.

In the present study, ED consumption decreased ALP levels. In studies conducted with athletes, it has been reported that ED consumption significantly reduces ALP serum levels^(29)^. The results of those studies are consistent with the results of the present study. In a study conducted in rats, it was reported that ED consumption significantly increased the serum ALP level^(22)^. The results of that study are different from those of the present study. In addition, in the present study, ED consumption caused a decrease in serum ALB/GLOB value and an increase in GLU value. In rat studies, it has been reported that ED causes an increase in glucose levels^(23)^.

In terms of hematological parameters, at the end of the 21st day, a significant increase in the number of LYM in all three groups, a significant increase in RDW-SD (fL) in the 20% ED group and a significant increase in PCT (mL/L) in the 10% ED group were observed. All these increases were above the reference value. On the other hand, there was a decrease in WBC (10^9^/L), MCV (fL) values and an increase in MCHC (g/dL), RDW-CV, PLT (10^9^/L), PCT (mL/L) values in the 10% ED group compared to the other groups. MPV (fL) increased in the 20% ED group. In addition, RBC (10^12^/L), HGB (g/Dl), HCT (%), MCV (fL) values in both treatment groups were higher than were the values in the control group depending on the ED consumption rate. These results are consistent with the results obtained in Khayyat et al.’s^(30)^ study (2014), in which the rats were given Red Bull ED.

In the present study, no significant differences were observed in cardiac, kidneys and brain index results of the female rats, while the differences between the groups in terms of utero-ovarian and liver index were significant (p < 0.05). Schuchowsky et al.^(31)^ reported that feeding male rats at different doses of Red Bull did not cause significant differences in liver, spleen and kidney weights. Similarly, Al-Mayyahi et al.^(23)^ reported that chronic ED intake did not affect liver and pancreas weights. In addition, in a different study, it was reported that ED consumption had an effect on kidney weight^(30)^. The results of the present study are consistent with the results of Qassim et al.’s study^(32)^, but differ from the results of Al-Mayyahi et al.’s^(23)^ and Schuchowsky et al.’s^(31)^ studies. It is thought that this difference may be due to the age and sex of the rats used in the present study, and due to the fact that the ED used in the study was sugar-free.

At the end of the present study, the differences determined between the offspring yields of the pregnant rats were significant. While similarity was observed between the control group and 10% ED group, the differences between the 20% ED group were significant. Consumption of high amounts of ED for a long time caused a decrease in the number of offspring. In a similar study, it was reported that energy drink administration caused atrophic and cystic ovarian changes and may cause infertility in the younger population consuming these drinks excessively^(33)^. In a different study, it was reported that high-dose caffeine consumption in mother rats affected the early stages of follicle development and had a permanent effect on folliculogenesis^(34)^.

Lakin et al.^(35)^ reviewed the effects of caffeine intake during pregnancy on human fetal development and reported that caffeine increased fetal respiration and heart rate and might increase lower growth and lower birth weight. They also reported that caffeine did not reduce the duration of pregnancy and did not cause hypertension, but caffeine increased uterine contractions and can potentially cause spontaneous abortion.

In a cohort study involving 4559 pregnancies, Ding et al.^(36)^ found that consumption of energy drinks before and during pregnancy was not associated with pregnancy loss, preterm birth, gestational diabetes, pre-eclampsia, while pre-pregnancy energy drink use was associated with a higher risk of gestational hypertension. Although the consumption of energy drinks cannot be considered clearly safe, serious concerns and uncertainties remain about their effects for women and fetus before and during pregnancy. The current study is expected to contribute to the literature and to clarify the effects of energy drinks in depth.

Beyond their physiological implications, the results of the present study offer critical clinical implications for midwifery and nursing practices, health care, and education. Healthcare professionals can directly utilize these result in providing preconception counseling and prenatal care to young women and expectant mothers to educate them about the potential adverse effects of energy drink consumption on fertility, maternal dietary behavior, and fetal development. In particular, the reduction in maternal nutrient intake and impaired postnatal weight gain in infants indicate that clinical nurses should closely monitor the nutritional status and infant growth trajectories of mothers who consume energy drinks in large amounts. Additionally, these biological markers provide a strong foundation for health research, enabling the design of observational studies and the development of targeted public health campaigns addressing modern dietary risks during the perinatal period.

## CONCLUSION

Thanks to the experimental animal model established using newly weaned rats, in the present study, it was determined that pre-pregnancy ED consumption increased water and feed intake in female rats; however, it still increased fluid intake but reduced feed intake during pregnancy. It was concluded that ED consumption before pregnancy and at the end of pregnancy increased Lee’s body mass index towards obesity. In the present study, it was observed that ED consumption, depending on the dose, increased serum creatinine levels, affected total cholesterol levels according to the amount of what was consumed, increased total protein levels, and reduced alkaline phosphatase levels. These findings serve as an evidence base for midwives and nurses when they counsel pregnant patients and those planning pregnancy on the potential risks of excessive energy drink consumption, thereby promoting healthier reproductive outcomes.

We recommend that further studies should be conducted both on experimental animal models and on humans, regarding the effects of energy drinks before pregnancy, during pregnancy, and on the fetus and newborn. Energy drinks have emerged as a passion and necessity for humanity, and sharing these results could help to address the concerns that they raise in society and among consumers, as well as informing the development of new products. Furthermore, it is believed that sharing the results of the present study and similar studies could benefit producers by helping them to eliminate the doubts and hesitations surrounding energy drink consumption.

## Data Availability

The data and analyses from this study will be made available by the authors upon reasonable request for academic and scientific review purposes. Data sharing will be conducted in accordance with legal requirements and ethical principles regarding the protection of personal data.
