# Supplementary material for: Effects of energy drinks on fertility, blood parameters and perinatal physiology in female rats
Source: Rev Esc Enferm USP. 2026 Aug 31;60:e20260353. doi: 10.1590/1980-220X-REEUSP-2026-0353en (PMC13551603; doi:10.1590/1980-220X-REEUSP-2026-0353en)
Supplement: Translation

# Translation: Efectos de las bebidas energéticas sobre la fertilidad, los parámetros sanguíneos y la fisiología perinatal en ratas hembras

**DOI:** 10.1590/1980-220X-REEUSP-2026-0353es

**Authors:** Halime Aydemir, Fatma Ergün, Ayşegül Turan

**Affiliations:** 1 Kirsehir Ahi Evran University, Faculty of Health Sciences, Department of Midwifery. Kirsehir, Türkiye.; 2 Kirsehir Ahi Evran University, Faculty of Health Sciences, Department of Nutrition and Dietetics, Kirsehir, Türkiye.; 3 Kirsehir Ahi Evran University, Faculty of Health Science, Department of Nursing. Kirsehir, Türkiye.

**Keywords:** Cafeína, Bebidas Energéticas, Fertilidad, Atención Perinatal, Ratas.

## Abstract

**Objetivo:**

El creciente consumo de bebidas energéticas, especialmente entre niños y jóvenes, en los últimos años genera preocupación. El objetivo de este estudio fue investigar los efectos del consumo de bebidas energéticas sobre la fertilidad, los parámetros sanguíneos y la fisiología perinatal en ratas hembras.

**Método:**

Se formaron tres grupos. De ellos, uno fue el grupo de control y los otros dos fueron los grupos experimentales, a saber, el grupo con 10 % de BE y el grupo con 20 % de BE. En cada grupo había 9 ratas (3 ratas por jaula, 3 jaulas de cría por grupo). A las ratas del grupo de control se les dio únicamente agua del grifo; a las del grupo con 10 % de bebidas energéticas se les dio una mezcla que incluía un 10 % de bebidas energéticas y un 90 % de agua del grifo; y a las del grupo con 20 % de bebidas energéticas se les dio una mezcla que incluía un 20 % de bebidas energéticas y un 80 % de agua del grifo.

**Resultados:**

El consumo de bebidas energéticas antes del embarazo aumentó la ingesta de agua y alimento en las ratas hembras; sin embargo, durante el embarazo siguió aumentando la ingesta de líquidos, pero redujo la ingesta de alimento. La diferencia entre los grupos fue estadísticamente significativa (p < 0,05). El consumo de bebidas energéticas afectó los parámetros hematológicos en función de la dosis, aumentó los niveles séricos de creatinina, afectó los niveles de colesterol total según el consumo, aumentó los niveles de proteína total y disminuyó los niveles de fosfatasa alcalina. El consumo de bebidas energéticas también redujo el número de crías y el aumento de peso posnatal de manera dependiente de la dosis.

**Conclusión:**

Se concluyó que las bebidas energéticas tuvieron efectos en las ratas hembras durante los períodos prenatal y perinatal. Para investigar los efectos potencialmente preocupantes del consumo de bebidas energéticas e identificar los posibles problemas de salud a largo plazo que podrían causar, se deben realizar más estudios en animales y en humanos. Estos resultados proporcionan evidencia biológica fundamental para ayudar a las parteras, las enfermeras especializadas en salud de la mujer y las enfermeras de salud pública a diseñar programas de asesoramiento premarital y educación prenatal basados en la evidencia, en los que se aborden los riesgos del consumo de bebidas energéticas para la fertilidad, la nutrición materna y el desarrollo neonatal.

## INTRODUCCIÓN

Los seres humanos se desarrollan y cambian continuamente a lo largo de sus vidas. Durante este proceso, influyen en su entorno y, a su vez, este influye en ellos. Para los seres humanos, en particular, las primeras etapas de la vida, como la infancia, la adolescencia y la juventud, revisten gran importancia. Una nutrición inadecuada y desequilibrada durante estos períodos puede afectar las etapas posteriores de la vida. Una alimentación saludable durante las primeras fases de la vida puede ayudar a reducir posibles consecuencias negativas. En los últimos años, los cambios en los patrones alimentarios han llevado a una diversificación de los alimentos y bebidas que consumimos. La nutrición en los seres humanos se encuentra entre los factores clave que afectan directamente su salud reproductiva y fertilidad al influir en el sistema endocrino^(1)^.

Las bebidas constituyen una parte significativa de nuestra dieta diaria. El líquido más consumido en el mundo es el agua. Las necesidades de líquidos del cuerpo de una persona pueden calcularse como 1 mL de líquido por cada caloría consumida al día o 30 mL de líquido por kilogramo. La Autoridad Europea de Seguridad Alimentaria (EFSA) indica que el valor de referencia de la ingesta total de líquidos es de 2,5 L para los hombres y de 2 L para las mujeres^(2)^. La variedad y la cantidad de líquidos que se consumen como bebidas aumentan día a día.

Como resultado de los avances en el sector alimentario, se ha producido un aumento significativo en el consumo de cafeína y un incremento en la demanda de bebidas con cafeína^(3)^. El aumento tanto en la cantidad como en la variedad de la ingesta diaria de líquidos se ha asociado con la aparición de diversos problemas de salud. Entre estos problemas de salud, los trastornos relacionados con la infertilidad ocupan un lugar importante. En varios estudios se ha indicado que reducir o evitar el consumo de cafeína puede mejorar la fertilidad tanto en mujeres como en hombres^(4)^. La cafeína está presente en grandes cantidades, especialmente en el café, el té y las bebidas energéticas, cuyo consumo ha aumentado en los últimos años^(5)^. A nivel mundial, el consumo de bebidas energéticas (BE) ha ido en constante aumento. Estas bebidas son consumidas comúnmente por adolescentes y adultos jóvenes. Se informa que más del 50% de las bebidas energéticas son consumidas por personas de estos grupos de edad^(6)^. Además, en un estudio realizado por Seifert et al.^(7)^, se informó que el 31% de los adolescentes de entre 12 y 17 años, y el 34% de los adultos jóvenes de entre 18 y 24 años, consumían regularmente bebidas energéticas.

Las bebidas energéticas se diferencian de otras bebidas que contienen cafeína por su composición química. Los ingredientes principales de estas bebidas incluyen cafeína, taurina, guaraná, azúcar, sodio y vitamina B6^(8)^. Además, ciertas bebidas energéticas producidas por fabricantes específicos también contienen componentes como glucuronolactona, ginseng y ginkgo biloba^(9)^.

En varios estudios recientes se han investigado los efectos a corto y largo plazo de las bebidas energéticas en el sistema cardiovascular y el sistema nervioso central. Según lo informado en estos estudios, las bebidas energéticas causan diversos trastornos metabólicos, como hipertensión arterial, enfermedades cardiovasculares, trastornos renales y trastornos del sueño en los seres humanos, especialmente en los jóvenes^(10)^. Se sabe que las bebidas energéticas aumentan la fatiga después de un cierto período de consumo y que su consumo se incrementa cuando se combinan con otras bebidas. También se ha informado que reducen el consumo de agua y la salivación, y que provocan erosión dental. Asimismo, se sabe que causan trastornos nerviosos, espasmos de las arterias coronarias y paro cardíaco en los jóvenes debido al consumo excesivo^(11)^. Además, en estudios experimentales realizados con modelos animales, se ha informado que el consumo de bebidas energéticas causa trastornos histopatológicos en diversos órganos, incluidos el cerebro, el hígado, los riñones y el corazón; conduce a la obesidad y aumenta los niveles de colesterol y glucosa en sangre^(12)^.

En los últimos años, la creciente prevalencia de trastornos de infertilidad entre los jóvenes ha llevado a comprender la importancia del tema y a investigar las causas subyacentes. Dado que las parteras y las enfermeras se encuentran entre los principales profesionales de la salud que brindan educación y asesoramiento en materia de salud a las mujeres en edad reproductiva, comprender los efectos fisiológicos de las bebidas energéticas es esencial para desarrollar estrategias efectivas de educación para las pacientes. En una revisión de la literatura, se reveló que el número de estudios en los que se investigan los efectos de las bebidas energéticas sobre el embarazo y la fertilidad es muy reducido. Los efectos del consumo de bebidas energéticas durante el embarazo aún no están claros. En el presente estudio, se estableció un modelo experimental en animales utilizando ratas recién destetadas. Con este modelo, se buscó investigar los efectos de las bebidas energéticas sobre la fertilidad, los parámetros sanguíneos y la fisiología perinatal en ratas hembras.

## MÉTODO

Las ratas hembras de 20 días de edad (Wistar albino) utilizadas en el estudio fueron criadas en la Unidad de Experimentos con Animales de la Facultad de Medicina de la Universidad Ahi Evran de Kirsehir. Las ratas se mantuvieron en un ciclo artificial de 12 horas de luz y 12 horas de oscuridad (luces encendidas entre las 7:00 a. m. y las 7:00 p. m.) a una temperatura constante de 22 ± 2 °C y una humedad relativa del 65%. Las ratas utilizadas en el estudio fueron alimentadas ad libitum. Para la alimentación de las ratas se utilizó una dieta comercial estándar para roedores de laboratorio (OPTIMA) (24% de proteína bruta, 3,94% de celulosa bruta, 5,08% de grasa bruta, 8,8% de ceniza bruta, 1,44% de lisina, 0,61% de metionina, 1,14% de calcio, 0,89% de fósforo, 0,28% de sodio). Antes de iniciar los experimentos, el protocolo de uso de animales fue aprobado por el comité de ética local para experimentos con animales de la Universidad Ahi Evran de Kirsehir (Fecha: 06/03/2025, N.º: 5/7). Todos los métodos y detalles utilizados en el estudio se llevaron a cabo de acuerdo con los criterios especificados en las directrices ARRIVE. Además, en todos los procedimientos aplicados a animales vivos en el estudio, se cumplió con el Reglamento sobre el Bienestar y la Protección de los Animales Utilizados con Fines Experimentales y Otros Fines Científicos del Ministerio de Alimentación, Agricultura y Ganadería de la República de Turquía^(13)^.

Se utilizaron los procedimientos establecidos por el Comité de Ética Local de la Universidad Ahi Evran de Kirsehir para monitorear la salud y el bienestar de los animales utilizados en el estudio. Con este fin, se monitoreó diariamente a los animales utilizados en el estudio hasta el final del mismo para detectar cambios tales como pérdida de peso, muerte, cambios de comportamiento, disminución en la ingesta de alimentos y agua, infección, abscesos, deshidratación, desnutrición, debilidad general, diarrea, estreñimiento, íleo, convulsiones y coma^(13,14)^.

No se observaron signos de malestar o enfermedad en ningún animal durante ningún período del estudio, y no se aplicaron criterios de eutanasia precoz. En casos tales como trastornos traumáticos causados por razones humanas, pérdida de peso superior al 25% del peso corporal, incapacidad para caminar adecuadamente, ingesta inadecuada de alimentos o agua, o disminución significativa de la respuesta a los estímulos, según lo establecido en la directiva del Comité de Ética Local de la Universidad Ahi Evran de Kirsehir (14) relativa al procedimiento de “Criterios para la exclusión de animales del protocolo de investigación”, si el veterinario lo considera apropiado, el animal o los animales se retiran del estudio y se les practica la eutanasia mediante la administración de dosis elevadas de xilazina (10 mg/kg de peso vivo, por vía intraperitoneal) y ketamina (60 mg de clorhidrato de ketamina/kg de peso vivo, por vía intraperitoneal).

### Ingredientes de las Bebidas Energéticas

En el estudio se utilizó la bebida energética sin azúcar de la marca Red Bull/Sugarfree^®^, que es ampliamente consumida por los jóvenes. Esta bebida energética se vende en latas de 250 mL y contiene cafeína (150 mg/L), taurina (800 mg/L), vitaminas (Nisain (8 mg/100 mL), ácido pantoténico (2 mg/100 mL), B6 (2 mg/100 mL), B12 (2 µg/100 mL), saborizantes y espesantes (goma xantana), colorantes (caramelo natural, riboflavina) y fenilalanina. La razón por la que se optó por la bebida energética sin azúcar fueron los efectos relacionados con el consumo de carbohidratos y la idea de que estos efectos podrían enmascarar otros resultados. La cantidad de bebida energética que se les administraría a las ratas se calculó considerando que un adulto de 65 kg podría cubrir el 10% y el 20% de la cantidad total de líquido que debería consumir si bebiera una lata de bebida energética (250 mL) y dos latas (250 mL + 250 mL), respectivamente, al día^(15)^. Los ingredientes de las bebidas energéticas varían poco entre las distintas marcas. Por lo tanto, las bebidas energéticas suelen contener ingredientes similares (cafeína, taurina, glucuronolactona, azúcar, vitaminas del grupo B y extractos de plantas [ginseng, guaraná, yerba mate y té verde]).

### Grupos de Estudio

Basándose en estudios publicados similares, se realizó un análisis de poder estadístico utilizando G*Power y el paquete de software estadístico MINITAB^(16)^. Como resultado de este análisis, se decidió incluir un mínimo de nueve animales por grupo para lograr una potencia estadística adecuada, lo que dio como resultado un total de 27 animales ^(16,17)^. Se formaron tres grupos con 9 ratas en cada uno (3 ratas en 1 jaula, tres jaulas de cría en cada grupo) (un total de 27 ratas). Mientras que a las ratas del grupo de control (C) solo se les proporcionó agua del grifo, a las ratas del grupo con 10% de ED se les administró una mezcla compuesta por un 90% de agua del grifo y un 10% de ED, y a las ratas del grupo con 20% de ED se les administró una mezcla compuesta por un 80% de agua del grifo y un 20% de ED, ad libitum durante todo el estudio (desde el primer día del estudio hasta que las ratas dieron a luz).

### Procedimiento Experimental

El experimento se llevó a cabo en dos fases durante un total de 54 días. La primera fase duró 21 días y se administró BE a los grupos en las proporciones determinadas. Al cabo de 21 días, se practicó una incisión en la región abdominal de 3 ratas con un peso corporal de 113,33 ± 1,52 g de cada grupo, bajo anestesia general con xilazina (10 mg/kg de peso corporal, por vía intraperitoneal) y ketamina (60 mg de clorhidrato de ketamina/kg de peso corporal, por vía intraperitoneal), y en condiciones de asepsia y antisepsia. Se realizó una punción en la aorta abdominal a las ratas antes de su muerte. Además, se extrajeron tejidos uterino-ovarianos, renales, hepáticos, cardíacos y cerebrales para el cálculo de los índices orgánicos. En la segunda fase del estudio, se permitió que las 6 ratas restantes de cada grupo quedaran preñadas. Se mantuvieron dos ratas hembras y una rata macho en jaulas de apareamiento durante 6 días. Posteriormente, las ratas hembras se colocaron en jaulas de cría (425 × 265 × 150/800 cm^2^), con dos ratas en cada jaula. Todas las ratas permanecieron en estas jaulas hasta el final del estudio (Figura 1).

**Figura 1:** Esquema general del diseño del estudio.

### Análisis

#### Análisis Físicos

***Consumo de alimento y agua e índice de masa corporal:*** Se midieron las cantidades de alimento y agua consumidas por las ratas a intervalos de 3 días, y al final del estudio se determinaron las cantidades de agua (mL/100 g de peso corporal) y alimento (g/100 g de peso corporal) consumidas por las ratas. Se midieron el peso corporal (PC) (con la báscula de precisión NECK WT-NF) y la longitud corporal (longitud nasoanal) (cm) (la distancia desde el hueso nasal hasta el ano cuando las ratas estaban acostadas boca abajo). El índice de masa corporal de los grupos de estudio se calculó en los días 0, 10, 21 y 40 utilizando la siguiente fórmula^(18)^.


$$\text{Indice de masa de Lee}=\frac{\sqrt[{3}]{\text{Peso corporal (g)}}}{\text{Altura (cm)}}\times 1000$$

### Análisis de la Función Motora

***Prueba de la rejilla invertida de Kondziela (Kondziela’s inverted screen test- KIST):*** La prueba de la rejilla invertida de Kondziela es una prueba de fuerza muscular que utiliza las cuatro extremidades. Para ello, se utilizó un aparato de plástico de 30 × 30 cm con orificios en forma de rejilla de 2 × 2 cm, diseñado especialmente para el estudio. En primer lugar, se colocó a las ratas en el centro del aparato. Luego se las colocó boca abajo desde una altura de 40 a 50 cm. Cuando la cabeza de la rata se inclinaba hacia abajo, se iniciaba un cronómetro y se medía el tiempo que las ratas permanecían suspendidas.

***Prueba de peso (Weight Test – WT):*** Con esta prueba se midió la fuerza muscular de las extremidades delanteras de las ratas. Se utilizaron pesas de plomo (gr) sujetas a una bola hecha de hilo de tejer como aparato de pesas^(19)^. A cada rata se la sujetó por la mitad de la cola y se la bajó para que agarrara la bola. Una vez que las ratas agarraron la bola con sus patas delanteras, se las levantó hacia arriba para que levantaran el peso que colgaba de la bola junto con esta. Si la rata dejaba caer el peso en menos de 3 segundos, se realizaba un segundo intento después de 10 segundos. Si fallaba tres veces, se daba por terminado este intento y se determinaba el peso máximo que la rata podía levantar.

### Análisis de Sangre y Suero

El día 21 del estudio, se recolectaron muestras de sangre a través de la aorta abdominal y se colocaron en tubos con ácido etilendiaminotetraacético (EDTA) para el análisis hematológico. Para obtener suero a partir de las muestras de sangre recolectadas para los parámetros bioquímicos, se centrifugó un total de 1,5 ml de sangre (2500 rpm, 10 min, 4 °C). Se determinaron las concentraciones séricas de creatinina (CRE), calcio (Ca), gamma-glutamiltransferasa (GGT), colesterol total (TCHO), proteína total (TP), fosfatasa alcalina (ALP), relación albúmina/globulina (ALB/GLOB) y glucosa (GLU) en las muestras de suero de ratas hembras se determinaron utilizando kits de análisis comerciales en un analizador FUJIFILM DRI-CHEM NX600 (Hasvet®). Además, utilizando el dispositivo Mindray BC-30 Vet (Hasvet®), se realizaron análisis de parámetros hematológicos a partir de muestras de sangre con EDTA, incluyendo glóbulos blancos (WBC), linfocitos (LYM), monocitos (MID), granulocitos (GRAN), relación neutrófilos-linfocitos (NLR), relación plaquetas-linfocitos (PLR), glóbulos rojos (RBC), hemoglobina (HGB), hematocrito (HCT), volumen corpuscular medio (MCV), hemoglobina corpuscular media (MCH), concentración de hemoglobina corpuscular media (MCHC), amplitud de distribución de los glóbulos rojos (RDW-CV), recuento de plaquetas (PLT), volumen plaquetario medio (MPV), amplitud de distribución plaquetaria (PDW), procalcitonina (PCT), índice de plaquetas de células grandes (PLCR) y coeficiente de plaquetas de células grandes (P-LCC).

### Análisis Anatómicos

El día 21 del estudio, se diseccionaron rápidamente el complejo útero-ovárico, el corazón, el hígado, el cerebro y los riñones obtenidos de las ratas sometidas a procedimientos quirúrgicos. Se retiraron el tejido conectivo y la fascia, y se secaron con papel de filtro los fluidos superficiales presentes en los órganos. Posteriormente, se pesaron los órganos. A continuación, se calcularon los índices de los órganos utilizando la siguiente fórmula^(20)^.


$$\text{Índice de órganos (\%)}=\frac{\text{Peso de órgano (g)}}{\text{Peso corporal total (g)}}\times 100$$

### Análisis de los datos de Fertilidad y Descendencia

En la segunda fase del estudio, se monitoreó el parto de las ratas hembras que habían sido separadas de los machos después del apareamiento y alojadas en jaulas de cría. Al final del período de observación, se registraron el número de hembras que dieron a luz en cada grupo, el número de crías, el peso al nacer de las crías y el aumento de peso diario de las crías hasta el día 10.

### Efectos Adversos

No se identificaron complicaciones relacionadas con el consumo de bebidas energéticas en las ratas de este estudio. No se observaron signos clínicos ni patológicos en los animales durante el período de estudio.

### Análisis Estadísticos

Los datos obtenidos del estudio se analizaron utilizando el SPSS (Statistical Package for the Social Sciences), versión 22. Inicialmente, se determinaron los efectos del consumo de bebidas energéticas sobre las características físicas y fisiológicas de las ratas jóvenes, preñadas y de las crías mediante un análisis de varianza unidireccional (ANOVA). Cuando se observaron diferencias significativas entre los grupos, se aplicó la prueba de comparaciones múltiples de Duncan para identificar el tratamiento o los tratamientos específicos que dieron lugar a estas variaciones. Para todas las evaluaciones estadísticas realizadas en este estudio, se consideró estadísticamente significativo un p-valor inferior a 0,05 (p < 0,05).

### Aprobación Ética y Consentimiento para Participar

Se obtuvo la autorización del comité de ética local de la Universidad Ahi Evran de Kirsehir para experimentos con animales, con fecha del 6 de marzo de 2025 y número 5/7. Todos los métodos y detalles utilizados en el estudio se llevaron a cabo de acuerdo con los criterios especificados en las directrices ARRIVE. Se utilizaron los procedimientos establecidos por el Comité de Ética Local de la Universidad Ahi Evran de Kirsehir para monitorear la salud y el bienestar de los animales utilizados en el estudio.

## RESULTADOS

En el estudio, se calculó el consumo diario de alimento y agua de las ratas hembras previas al apareamiento y de las ratas gestantes en cada grupo. Las diferencias entre los grupos fueron estadísticamente significativas a un nivel de p < 0,05. El mayor consumo de agua se observó en los grupos con una dieta restringida al 10% (BE). El mayor consumo de alimento se registró en el grupo con una dieta restringida al 20% (BE) entre las ratas hembras previas al apareamiento, y en el grupo C entre las ratas gestantes. En el estudio, se midieron el peso corporal y la longitud corporal de las ratas hembras de los grupos en los días 0, 10, 21 y 40, y se calcularon sus valores del índice de masa corporal. Las diferencias en los valores del índice de masa corporal entre los grupos fueron significativas a un nivel de p < 0,05 (Tabla 1). Se determinó que el consumo de BE antes y durante el embarazo aumentó los valores del índice.

**Tabla 1:** Consumo diario de ración y agua y resultados del índice de masa corporal de los grupos – Kirsehir, Turquía, 2026.

Grupos	Cantidades diarias de consumo de ración y líquido de los grupos						
	Consumo de ración de las ratas hembras antes del apareamiento (g/100 g de peso corporal/día)		Consumo de agua/líquidos de las ratas hembras antes del apareamiento (mL/100 g de peso corporal/día)		Consumo de ración de las ratas gestantes (g/100 g de peso corporal/día)		Consumo de agua/líquidos de las ratas gestantes (ml/100 g de peso corporal/día)
C	0,13 ± 0,51^b^		0,45 ± 0,04^b^		0,17 ± 0,05^a^		0,50 ± 0,03^b^
10 % BE	0,14 ± 0,34^a^		0,58 ± 0,03^a^		0,16 ± 0,01^a^		0,54 ± 0,01^a^
20 % BE	0,14 ± 0,80^a^		0,48 ± 0,03^b^		0,16 ± 0,01^a^		0,49 ± 0,01^b^
**Grupos**	**Valores del índice de masa de Lee de los grupos en diferentes intervalos de tiempo**						
	**Día 0**		**Día 10**		**Día 21**		**Día 40**
C	293,70 ± 6,98^a^		285,82 ± 0,45^b^		289,04 ± 6,16^b^		288,00 ± 6,03^b^
10 % BE	292,69 ± 2,43^a^		296,55 ± 0,61^a^		298,00 ± 2,84^a^		296,28 ± 5,73^a^
20 % BE	290,40 ± 4,30^a^		295,98 ± 0,38^a^		297,13 ± 3,59^a^		294,80 ± 4,86^ab^

*: Las diferencias entre las medias indicadas con la misma letra no son significativas a un nivel de p < 0,05; C: al grupo de control se le administró agua del grifo; al grupo con un 10 % de bebida energética se le administró una mezcla compuesta por un 10 % de bebida energética y un 90 % de agua del grifo; al grupo con un 20 % de bebida energética se le administró una mezcla compuesta por un 20 % de bebida energética y un 80 % de agua del grifo.

En el estudio, se evaluaron las funciones motoras de las ratas antes del embarazo mediante dos paradigmas. Se midió la fuerza muscular de las cuatro extremidades de las ratas con la prueba de la rejilla invertida de Kondziela. Las diferencias entre los grupos fueron significativas (p < 0,05). Además, el valor más alto en la prueba de peso entre los grupos se observó en el grupo con 20% de BE (p < 0,05) (Figura 2).

**Figura 2:** Resultados de la prueba de la rejilla invertida de Kondziela y de la prueba de peso aplicadas a los grupos.

KIST: Prueba de rejilla invertida de Kondziela; WT: Medida de peso; C: Al grupo de control se le administró agua del grifo; al grupo BE al 10 % se le administró una mezcla compuesta por un 10 % de bebida energética y un 90 % de agua del grifo; al grupo BE al 20 % se le administró una mezcla compuesta por un 20 % de bebida energética y un 80 % de agua del grifo.

El día 21 del estudio se analizaron las muestras de suero sanguíneo extraídas de las ratas. Las diferencias entre los grupos en cuanto a los niveles de CRE, TCHO, TP, ALP, ALB/GLOB y GLU fueron estadísticamente significativas (Tabla 2) (p < 0,05). En el estudio se realizaron análisis sanguíneos completos a ratas hembras antes del apareamiento y se determinaron los resultados de los parámetros hematológicos para cada grupo. Se observaron diferencias estadísticamente significativas entre los grupos en cuanto a los parámetros WBC, RBC, HGB, HCT, MCV, MCHC, RDW-CV, PLT, MPV, PCT y P-LCC (p < 0,05) (Tabla 2).

**Tabla 2:** Valores de los parámetros sanguíneos de los grupos – Kirsehir, Turquía, 2026.

Parámetros	Valores de los parámetros bioquímicos de los grupos			
	C	10 % BE	20 % BE	Valor de referencia
CRE	0,28 ± 0,05^b^	0,31 ± 0,01^a^	0,33 ± 0,02^a^	–
Ca	4,36 ± 0,32^a^	4,70 ± 0,55^a^	4,83 ± 0,15^a^	–
GGT	10,66 ± 1,15^a^	11,33 ± 2,30^a^	10,33 ± 0,57^a^	–
TCHO	52,33 ± 4,04^ab^	56,66 ± 0,57^a^	50,33 ± 0,57^b^	–
TP	5,93 ± 0,28^b^	6,66 ± 0,11^a^	6,00 ± 0,20^b^	–
ALP	5,26 ± 0,57^a^	4,50 ± 0,10^ab^	4,13 ± 0,49^b^	–
ALB/GLOB	2,76 ± 0,57^a^	1,93 ± 0,20^b^	1,96 ± 0,11^b^	–
GLU	134,66 ± 2,30^b^	178,00 ± 8,66^a^	172,00 ± 15,58^a^	–
	**Valores de los parámetros hematológicos de los grupos**			
Leucocitos (10^9^/L)	11,29 ± 1,38^ab^	10,59 ± 0,24^b^	13,16 ± 1,53^a^	1.90–13.80
Granulocitos (10^9^/L)	3,16 ± 0,32^a^	2,40 ± 0,75^a^	3,74 ± 0,90^a^	0.39–4.60
Linfocitos (10^9^/L)	**7,57 ± 1,08** ^a^	**7,24 ± 0,22** ^a^	**7,22 ± 1,76** ^a^	1.31–7.20
Mon (10^9^/L)	0,57 ± 0,02^b^	0,59 ± 0,06^b^	0,82 ± 0,15^a^	0.00–0.92
Gran (%)	0,26 ± 0,02^a^	0,28 ± 0,04^a^	0,27 ± 0,03^a^	0.120–0.460
Linfocitos (%)	0,68 ± 0,03^a^	0,66 ± 0,03^a^	0,66 ± 0,03^a^	0.500–0.859
Mon (%)	0,050 ± 0,008^a^	0,051 ± 0,008^a^	0,055 ± 0,01^a^	0.000–0.103
Glóbulos rojos (10^12^/L)	6,10 ± 0,35^b^	**7,49 ± 0,50** ^a^	**7,09 ± 0,53** ^a^	4.82–9.80
HGB (g/dl)	13,90 ± 0,17^b^	**16,73 ± 0,28** ^a^	**16,56 ± 0,75** ^a^	11.0–17.0
HCT (%)	39,00 ± 1,07^b^	**45,16 ± 0,40** ^a^	**45,36 ± 1,87** ^a^	27.0–46.0
VCM (fL)	64,10 ± 2,62^a^	58,73 ± 1,76^b^	62,50 ± 0,69^a^	50.0–67.0
HMC (pg)	22,86 ± 1,25^a^	21,70 ± 0,52^a^	22,26 ± 0,40^a^	16.0–25.0
MCHC (g/dL)	355,33 ± 6,42^b^	369,66 ± 2,51^a^	355,66 ± 2,30^b^	310–390
RDW-CV	0,15 ± 0,06^b^	0,11 ± 0,07^c^	0,17 ± 0,02 ^a^	0.110–0.150
RDW-SD (fL)	38,30 ± 1,87^b^	27,40 ± 1,21^c^	**42,56 ± 0,28** ^a^	25.0–40.0
PLT (10^9^/L)	872,33 ± 10,64^b^	1251,33 ± 2,88^a^	822,33 ± 40,47^b^	250–1400
MPV (fL)	6,63 ± 0,32^b^	6,43 ± 0,37^b^	7,23 ± 0,11^a^	5.1–8.2
PDW	15,30 ± 0,40^a^	14,96 ± 0,05^a^	15,30 ± 0,40^a^	12.0–17.5
PCT (mL/L)	5,96 ± 0,56^c^	**8,30 ± 0,09** ^a^	7,16 ± 0,56^b^	2.00–8.20
P-LCC (10^9^/L)	299,33 ± 27,13^a^	429,66 ± 11,54^a^	373,33 ± 13,27^a^	100–580
P-LCR	0,32 ± 0,02^c^	0,37 ± 0,02^b^	0,42 ± 0,09^a^	0.220–0.570

*: Las diferencias entre las medias indicadas con la misma letra en cada fila no son significativas a un nivel de p < 0,05; C: al grupo de control se le administró agua del grifo; al grupo 10 % BE se le administró una mezcla compuesta por un 10 % de bebida energética y un 90 % de agua del grifo; al grupo 20 % BE se le administró una mezcla compuesta por un 20 % de bebida energética y un 80 % de agua del grifo; CRE: creatinina; Ca: calcio; GGT: gamma-transferasa; TCHO: colesterol total; TP: proteína total; ALP: fosfatasa alcalina; ALB/GLOB: relación albúmina/globulina; GLU: glucosa; WBC: glóbulos blancos; LYM: linfocitos; MON: monocitos; GRAN: granulocitos; RBC: glóbulos rojos; HGB: hemoglobina; HCT: hematocrito; MCV: volumen corpuscular medio; MCH: hemoglobina corpuscular media; MCHC: concentración de hemoglobina corpuscular media; RDW-CV: amplitud de distribución de los glóbulos rojos; PLT: recuento de plaquetas; MPV: volumen plaquetario medio; PDW: amplitud de distribución plaquetaria; PCT: procalcitonina; PLCR: índice de plaquetas de células grandes; P-LCC: coeficiente de plaquetas de células grandes.

El día 21 del estudio se determinaron los índices de peso de los órganos de las ratas sometidas a cirugía. En el estudio, las diferencias entre los índices de los órganos cerebrales, renales y cardíacos no fueron significativas (p < 0,05). Sin embargo, las diferencias entre los índices de los tejidos uterino-ováricos y hepáticos sí fueron significativas. Al final del estudio, se determinó el número de crías de las ratas gestantes que dieron a luz en los distintos grupos, sus pesos y los pesos vivos de las crías al final del décimo día. Las diferencias entre los grupos en cuanto al número de crías nacidas y a los valores medios del peso vivo de las crías al final del décimo día fueron significativas (Tabla 3) (p < 0,05).

**Tabla 3:** Datos sobre los coeficientes de los órganos y las características de las crías de los grupos (%) – Kirsehir, Turquía, 2026.

Grupos	Datos sobre el coeficiente de órganos				
	Índice utero-ovárico	Índice corazón	Índice hepático	Índice de los riñones	Índice cerebral índice
C	0,44 ± 0,02^b^	0,41 ± 0,00^a^	4,69 ± 0,18^b^	0,95 ± 0,07^a^	0,95 ± 0,04^a^
10 % BE	0,55 ± 0,02^a^	0,42 ± 0,00^a^	5,27 ± 0,23^a^	0,93 ± 0,06^a^	0,92 ± 0,13^a^
20 % BE	0,37 ± 0,00^c^	0,40 ± 0,05^a^	4,38 ± 0,35^b^	0,10 ± 0,07^a^	0,98 ± 0,15^a^
**Grupos**	**Datos sobre la descendencia de los grupos**				
	**Número de crías (unidades)**		**Peso al nacer (gr)**		**Peso de las crías al décimo día (gr)**
C	12,75 ± 0,95^a^		5,89 ± 0,42^a^		18,94 ± 0,41^a^
10 % BE	11,75 ± 0,95^a^		6,24 ± 0,55^a^		18,62 ± 0,68^a^
20 % BE	10,75 ± 1,50^b^		6,48 ± 0,74^a^		16,70 ± 0,83^b^

*: Las diferencias entre las medias indicadas con la misma letra no son significativas a un nivel de p < 0,05; C: al grupo de control se le administró agua del grifo; al grupo “10 % BE” se le administró una mezcla compuesta por un 10 % de bebida energética y un 90 % de agua del grifo; al grupo “20 % BE” se le administró una mezcla compuesta por un 20 % de bebida energética y un 80 % de agua del grifo.

## DISCUSIÓN

En el estudio, se observó un aumento en el peso corporal de las ratas debido al consumo de BE. Adjene et al.^(21)^ informaron que el consumo a largo plazo de bebidas energéticas aumentó el peso corporal en su estudio con ratas. Mustafa et al.^(22)^ se propusieron evaluar los efectos secundarios de bebidas energéticas como Red Bull y Red Strong en diversos parámetros fisiológicos y características histológicas del hígado y los riñones en ratas albinas macho, y encontraron que el aumento de peso corporal se incrementó significativamente en los grupos que consumieron altas dosis de bebidas energéticas a largo plazo. Los resultados del presente estudio concuerdan con los del estudio de Adjene et al.^(21)^. Por otro lado, en un estudio con ratas, se informó que las ratas a las que se les administró bebidas energéticas durante 140 días experimentaron una pérdida significativa de peso corporal en comparación con las ratas del grupo de control^(23)^. Este resultado difiere del del presente estudio. Se cree que esta diferencia se debió probablemente a la diferencia en la duración del consumo de bebidas energéticas por parte de las ratas.

Aunque se han establecido diversos criterios estándar para la detección de la obesidad en humanos, la obesidad y el aumento de peso corporal, incluso en animales, se detectan mediante diversos métodos, como el índice de masa corporal de Lee. En el índice de masa corporal de Lee, los valores superiores a 310 se aceptan como un indicador de obesidad^(18)^. Los valores determinados en el presente estudio están por debajo de este valor. Los valores más altos se observaron en los grupos que consumieron bebidas energéticas, lo que sugiere que estas podrían tener el potencial de causar obesidad, y que el efecto de la cafeína y los saborizantes en las bebidas energéticas también podría provocar esta situación. La cafeína también puede provocar un aumento en el consumo de alimentos debido al insomnio^(24)^.

En el presente estudio, la resistencia y la fuerza muscular en los grupos que consumieron bebidas energéticas fueron mayores que en el grupo de control, probablemente debido a que los componentes químicos de las bebidas energéticas influyen en esta situación, ya que estas sustancias actúan como protectores antioxidantes al regular la capacidad de trabajo de los músculos, la resistencia aeróbica y el transporte de Ca^2+^ en los músculos. También desempeñan un papel importante como reguladores de la presión osmótica en los tejidos^(25)^.

En el presente estudio, se determinó que el consumo de bebidas energéticas, en función de la dosis, aumentó la cantidad de CRE sérica, que es un producto de degradación del metabolismo muscular; actividades como el ejercicio y la actividad física excesivas, así como un alto consumo de proteínas, incrementan esta cantidad. En un estudio de 12 semanas con ratas que consumieron bebidas energéticas, se reportaron niveles elevados de enzimas hepáticas, función renal alterada y resultados lipídicos anómalos^(26)^. En otro estudio, se reportaron cambios histopatológicos en los tejidos renales y hepáticos de ratas que consumían bebidas energéticas^(22)^. Los resultados obtenidos en ese estudio concuerdan con los obtenidos en el presente estudio.

En el presente estudio, no se detectó ninguna correlación entre los grupos en cuanto a la cantidad de consumo de bebidas energéticas y el nivel de TCHO sérico. El valor más alto se determinó en el grupo con un 10% de bebidas energéticas y el valor más bajo, en el grupo con un 20% de bebidas energéticas. Las diferencias entre los grupos pueden explicarse por el hecho de que el consumo de un 10% de BE aumenta el nivel de TCHO al incrementar el acetil-CoA, que es necesario para la síntesis de colesterol en el hígado de las ratas Wistar hembras^(27)^. En el presente estudio, el consumo de BE aumentó el TP sérico en las ratas hembras. En un estudio, se informó que el consumo de BE disminuyó los niveles de TP en ratas hembras, lo que podría deberse a una alteración en la síntesis de proteínas hepáticas como resultado de una disfunción hepática^(28)^.

En el presente estudio, el consumo de BE disminuyó los niveles de ALP. En estudios realizados con atletas, se ha informado que el consumo de BE reduce significativamente los niveles séricos de ALP^(29)^. Los resultados de esos estudios son con­sistentes con los resultados del presente estudio. En un estudio realizado en ratas, se informó que el consumo de BE aumentó significativamente el nivel sérico de ALP^(22)^. Los resultados de ese estudio difieren de los del presente estudio. Además, en el presente estudio, el consumo de BE provocó una disminución en el valor sérico de ALB/GLOB y un aumento en el valor de GLU. En estudios con ratas, se ha informado que el BE provoca un aumento en los niveles de glucosa^(23)^.

En cuanto a los parámetros hematológicos, al final del día 21 se observó un aumento significativo en el recuento de linfocitos (LYM) en los tres grupos, un aumento significativo en el RDW-SD (fL) en el grupo con 20% de BE y un aumento significativo en la PCT (mL/L) en el grupo con 10% de BE. Todos estos aumentos se situaron por encima del valor de referencia. Por otro lado, se observó una disminución en los valores de leucocitos (10^9/L) y del volumen corpuscular medio (MCV, fL), así como un aumento en los valores de concentración de hemoglobina en el plasma (MCHC, g/dL), del índice de variación de la distribución de la hemoglobina (RDW-CV), de plaquetas (PLT, 10^9/L) y de la proporción de plaquetas (PCT, mL/L) en el grupo con 10% de privación de alimentos, en comparación con los otros grupos. El MPV (fL) aumentó en el grupo que consumió un 20% de BE. Además, los valores de RBC (10^12^/L), HGB (g/dL), HCT (%), y MCV (fL) en ambos grupos de tratamiento fueron más altos que los del grupo de control, dependiendo de la tasa de consumo de BE. Estos resultados concuerdan con los obtenidos en el estudio de Khayyat et al.^(30)^, en el que se administró Red Bull BE a las ratas.

En el presente estudio, no se observaron diferencias significativas en los índices cardíacos, renales y cerebrales de las ratas hembras, mientras que las diferencias entre los grupos en cuanto a los índices uterino-ovariano y hepático fueron significativas (p < 0,05). Schuchowsky et al.^(31)^ informaron que alimentar a ratas macho con diferentes dosis de Red Bull no causó diferencias significativas en los pesos del hígado, el bazo y los riñones. De manera similar, Al-Mayyahi et al.^(23)^ informaron que la ingesta crónica de BE no afectó los pesos del hígado y el páncreas^(23)^. Además, en otro estudio, se informó que el consumo de BE tuvo un efecto sobre el peso de los riñones^(30)^. Los resultados del presente estudio concuerdan con los del estudio de Qassim et al.^(32)^ pero difieren de los resultados de los estudios de Al-Mayyahi et al.^(23)^ y de Schuchowsky et al.^(31)^. Se cree que esta diferencia puede deberse a la edad y el sexo de las ratas utilizadas en el presente estudio, así como al hecho de que la bebida energética utilizada en el estudio no contenía azúcar.

Al finalizar el presente estudio, las diferencias observadas en el número de crías de las ratas preñadas fueron significativas. Si bien se observó similitud entre el grupo de control y el grupo que consumió un 10% de bebida energética, las diferencias con respecto al grupo que consumió un 20% de bebida energética fueron significativas. El consumo de grandes cantidades de bebidas energéticas durante un período prolongado provocó una disminución en el número de crías. En un estudio similar, se informó que la administración de bebidas energéticas causó cambios ováricos atróficos y quísticos y podría provocar infertilidad en la población más joven que consume estas bebidas en exceso^(33)^. En otro estudio, se informó que el consumo de altas dosis de cafeína en ratas madres afectó las primeras etapas del desarrollo folicular y tuvo un efecto permanente en la foliculogénesis^(34)^.

Lakin et al.^(35)^ revisaron los efectos de la ingesta de cafeína durante el embarazo en el desarrollo fetal humano e informaron que la cafeína aumentaba la respiración y la frecuencia cardíaca del feto y podría estar relacionada con un menor crecimiento y un menor peso al nacer. También informaron que la cafeína no reducía la duración del embarazo ni causaba hipertensión, pero que aumentaba las contracciones uterinas y podía provocar un aborto espontáneo.

En un estudio de cohorte que incluyó 4559 embarazos, Ding et al.^(36)^ descubrieron que el consumo de bebidas energéticas antes y durante el embarazo no se asoció con la pérdida del embarazo, el parto prematuro, la diabetes gestacional ni la preeclampsia, mientras que el consumo de bebidas energéticas antes del embarazo se asoció con un mayor riesgo de hipertensión gestacional. Aunque el consumo de bebidas energéticas no puede considerarse claramente seguro, persisten serias preocupaciones e incertidumbres sobre sus efectos para las mujeres y el feto antes y durante el embarazo. Se espera que el presente estudio contribuya a la literatura científica y aclare a fondo los efectos de las bebidas energéticas.

Más allá de sus implicaciones fisiológicas, los resultados del presente estudio ofrecen implicaciones clínicas fundamentales para las prácticas de partería y enfermería, la atención de salud y la educación. Los profesionales de la salud pueden utilizar directamente estos resultados para brindar asesoramiento preconcepcional y atención prenatal a mujeres jóvenes y futuras mamás, con el fin de informarlas sobre los posibles efectos adversos del consumo de bebidas energéticas en la fertilidad, los hábitos alimenticios de la madre y el desarrollo fetal. En particular, la reducción en la ingesta de nutrientes maternos y el deterioro en el aumento de peso posnatal de los bebés indican que el personal de enfermería clínica debe monitorear de cerca el estado nutricional y las trayectorias de crecimiento de los bebés de las madres que consumen bebidas energéticas en grandes cantidades. Además, estos marcadores biológicos proporcionan una base sólida para la investigación en salud, lo que permite el diseño de estudios observacionales y el desarrollo de campañas de salud pública específicas que aborden los riesgos alimentarios modernos durante el período perinatal.

## CONCLUSIÓN

Gracias al modelo animal experimental establecido con ratas recién destetadas, en el presente estudio se determinó que el consumo de bebidas energéticas antes del embarazo aumentó la ingesta de agua y alimento en las ratas hembras; sin embargo, durante el embarazo siguió aumentando la ingesta de líquidos, pero redujo la ingesta de alimento. Se concluyó que el consumo de bebidas energéticas antes del embarazo y al final del mismo aumentó el índice de masa corporal de Lee hacia la obesidad. En el presente estudio, se observó que el consumo de bebidas energéticas, dependiendo de la dosis, aumentó los niveles séricos de creatinina, afectó los niveles de colesterol total según la cantidad consumida, aumentó los niveles de proteína total y redujo los niveles de fosfatasa alcalina. Estos hallazgos sirven como base de evidencia para las parteras y enfermeras cuando asesoran a pacientes embarazadas y a aquellas que planean un embarazo sobre los riesgos potenciales del consumo excesivo de bebidas energéticas, promoviendo así resultados reproductivos más saludables.

Recomendamos que se realicen más estudios, tanto en modelos animales experimentales como en humanos, sobre los efectos de las bebidas energéticas antes del embarazo, durante el embarazo y en el feto y el recién nacido. Las bebidas energéticas se han convertido en una pasión y una necesidad para la humanidad, y compartir estos resultados podría ayudar a abordar las inquietudes que generan en la sociedad y entre los consumidores, así como a orientar el desarrollo de nuevos productos. Además, se cree que compartir los resultados del presente estudio y de estudios similares podría beneficiar a los productores al ayudarlos a eliminar las dudas y vacilaciones en torno al consumo de bebidas energéticas.

## Data Availability

Los autores pondrán a disposición los datos y análisis de este estudio previa solicitud razonable con fines de revisión académica y científica. El intercambio de datos se llevará a cabo de conformidad con los requisitos legales y los principios éticos relativos a la protección de datos personales.
